# The Safety and Protective Efficacy Evaluation of an Attenuated *M. bovis*–BoHV-1 Bivalent Vaccine in Rabbits

**DOI:** 10.3390/vaccines11111698

**Published:** 2023-11-07

**Authors:** Sen Zhang, Yisheng Zhang, Guoxing Liu, Chen Wang, Yan Ji, Jianguo Chen, Changmin Hu, Xi Chen, Aizhen Guo, Yingyu Chen

**Affiliations:** 1 State Key Laboratory of Agricultural Microbiology, College of Veterinary Medicine, Huazhong Agricultural University, Wuhan 430070, China; zhangs416@webmail.hzau.edu.cn (S.Z.);; 2 Hubei International Scientific and Technological Cooperation Base of Veterinary Epidemiology, The Cooperative Innovation Center for Sustainable Pig Production, Wuhan 430070, China; 3 Key Laboratory of Development of Veterinary Diagnostic Products, Ministry of Agriculture and Rural Affair, Wuhan 430070, China; 4 Key Laboratory of Ruminant Biological Products, Ministry of Agriculture and Rural Affair, Hohhot 010011, China; 5 The Spirit JinYu Biological Pharmaceutical Co., Ltd., Hohhot 010030, China

**Keywords:** BRD, BoHV-1, *M. bovis*, attenuated vaccine, protective efficacy

## Abstract

Bovine respiratory disease (BRD) is a global prevalent multifactorial infection primarily caused by viral and bacterial coinfections. In China, *Mycoplasma bovis* (*M. bovis*) and bovine herpesvirus type 1 (BoHV-1) are the predominant pathogens associated with BRD. Our previous study involved the development of attenuated *M. bovis* HB150 and BoHV-1 gG-/tk- vaccine strains, which were thoroughly assessed for their safety profiles and protective efficacy in cattle. In this study, we applied a combination of vaccines in varying ratios and used a rabbit model to determine the safety and protective efficacy. We used PCR/RT-PCR to detect the postimmunization and challenge shedding of *M. bovis* and BoHV-1. Additionally, we measured antibody titers and the expression of IFN-β and TNF-α to evaluate the humoral and cellular immune responses, respectively. Furthermore, we performed a histopathological analysis to assess lung damage. Our study provides evidence of the safety and effectiveness of the bivalent *M. bovis*–BoHV-1 vaccine in rabbits, particularly when applying a combination of 1.0 × 10^8^ CFU of *M. bovis* HB150 and 1.0 × 10^6^ TCID50 of the BoHV-1 gG-/tk- strain. The bivalent vaccine significantly enhanced both the long-term antibody immune response and cellular protection against the *M. bovis* and BoHV-1 challenge. These findings provide a valuable model for the potential application in cattle.

## 1. Introduction

Bovine respiratory disease (BRD), which caused approximately 75% of illness and 50–70% of mortality in feedlots, stands as a widespread and economically challenging problem for the cattle industry [1,2]. BRD holds critical importance in dairy cattle during the pre- and postweaning periods [3]. More than 90% of large feedlots in the United States have reported BRD as the most prevalent disease, with an estimated annual cost ranging from USD 1 to 3 billion [4,5]. In Australia, the documented morbidity and mortality rates for BRD were 18% and 2.1%, respectively, with an average net loss of AUD 1647.53 per death [1]. BRD is a complex ailment influenced by various factors, such as stress, management practices, physiological conditions, and pathological agents [6,7,8]. Common pathogens associated with BRD include bovine herpesvirus type 1 (BoHV-1), bovine viral diarrhea virus (BVDV), bovine respiratory syncytial virus (BRSV), bovine parainfluenza virus type 3 (BPIV-3), *Pasteurella multocida*, *Mannheimia haemolytica*, and *Mycoplasma bovis* (*M. bovis*) [9].

*M. bovis* is currently the most prevalent causative agent of BRD globally. It can induce not only pneumonia, but also a variety of other conditions, including mastitis, arthritis, skin abscesses, meningitis, otitis, and reproductive tract disorders in cattle of all ages and across different sectors [10]. In China, *M. bovis* pneumonia was first reported in 2008 among newly imported beef cattle transported over long distances throughout the country, and has since become remarkably widespread [11]. To combat BRD caused by *M. bovis*, our laboratory developed a single attenuated vaccine using the clinical isolate *M. bovis* HB0801 strain in 2014. This vaccine exhibited a remarkable protection rate of 87.7% in cattle [12].

BoHV-1 is also an important causative agent of BRD in China. It was initially identified in an imported cow in 1981 and rapidly spread throughout the country. The overall prevalence of BoHV-1 among cattle in China is 40% [13]. In Inner Mongolia, the nucleic acid positive rate reached up to 24.83% [6]; in Henan province, it ranged from 7% to 15%; in Xinjiang, it was 9% [13]. Our previous study established a double-deleted strain, BoHV-1 gG-/tk-, which elicited robust immune protection in cattle [14].

Based on our previous work, we established rabbit infection models for *M. bovis* HB0801 and BoHV-1 HB06, respectively. Rabbits challenged by *M. bovis* HB0801 showed increased rectal temperature and respiratory symptoms, and lung consolidation was observed after dissection. The rectal temperature of rabbits challenged by BoHV-1 HB06 was immediately elevated and lasted for several days, followed by an increase in nasal secretions; histopathology observation of the lungs showed obvious lesions after dissection.

Single vaccines prove insufficient to address the challenges posed by mixed infections of multiple pathogens; therefore, we assumed a combination approach, using two vaccines with different doses based on the above studies. We evaluated its safety and efficacy in a rabbit model to establish its effect for the subsequent development of an attenuated *M. bovis*–BoHV-1 bivalent vaccine designed for calves.

## 2. Materials and Methods

### 2.1. Cells, Viruses, and the Culture of M. bovis and BoHV-1

Wild-type BoHV-1 HB06 (GenBank accession number: AJ004801.1), the BoHV-1 gG-/tk- strain, the *M. bovis* HB0801 (GenBank accession number: CP002058.1) strain, and the *M. bovis* HB150 strain were maintained in the National Key Laboratory of Agricultural Microbiology. Madin–Darby bovine kidney cells (MDBK) were obtained from the China Institute of Veterinary Drug Control.

*M. bovis* and BoHV-1 were cultured as previously reported [15,16]. Briefly, *M. bovis* HB0801 and HB150 strains were cultured in complete PPLO medium at 37 °C in a 5% CO_2_ incubator for 40–48 h. The BoHV-1 HB06 and BoHV-1 gG-/tk- strains were cultured using MDBK cells in Dulbecco’s modified Eagle’s medium (DMEM) supplemented with 10% fetal bovine serum (Opcel Biotechnology Co., Ltd., Hohhot, China) at 37 °C in a 5% CO_2_ incubator.

The initial dosages of the *M. bovis* HB150 and BoHV-1 gG-/tk- were 1.0 × 10^8^ CFU and 1.0 × 10^6^ TCID_50_, respectively. In this research, we mixed them in ratios of 1:1, 1:2, and 2:1, corresponding to the following combinations: 1.0 × 10^8^ CFU *M. bovis* HB150 with 1.0 × 10^6^ TCID_50_ BoHV-1 gG-/tk-, 1.0 × 10^8^ CFU *M. bovis* HB150 with 2.0 × 10^6^ TCID_50_ BoHV-1 gG-/tk-, and 2.0 × 10^8^ CFU *M. bovis* HB150 with 1.0 × 10^6^ TCID_50_ BoHV-1 gG-/tk-. The study objective was to determine the most effective combination of these two vaccine strains.

### 2.2. Animal Immunization and Challenge

Thirty-three Japanese white rabbits, with an average weight of 1.5 kg, were obtained from the Experimental Animal Center at Huazhong Agricultural University. The rabbits were tested for BoHV-1, *M. bovis*, *Pasteurella*, and *Manniella hemolysticus*, and all resulted negative. Subsequently, those rabbits were divided into 11 groups, each consisting of 3 rabbits. Groups 1 and 2, 3 and 4, and 5 and 6 were immunized with 1.0 × 10^8^ CFU *M. bovis* HB150 mixed with 1.0 × 10^6^ TCID_50_ of the BoHV-1 gG-/tk- strain (ratio of 1:1), 1.0 × 10^8^ CFU *M. bovis* HB150 mixed with 2.0 × 10^6^ TCID_50_ of the BoHV-1 gG-/tk- strain (ratio of 1:2), and 2.0 × 10^8^ CFU *M. bovis* HB150 mixed with 1.0 × 10^6^ TCID_50_ of the BoHV-1 gG-/tk- strain (ratio of 2:1), respectively; Group 7 was immunized with 1.0 × 10^6^ TCID_50_ of the BoHV-1 gG-/tk- strain; Group 8 was immunized with 1.0 × 10^8^ CFU of the *M. bovis* HB150 strain; Groups 9 and 10 were inoculated with DMEM and the complete PPLO medium, respectively. All experimental groups were then challenged with 1.0 × 10^9^ CFU of the *M. bovis* HB0801 strain or 4.0 × 10^7^ TCID_50_ of the BoHV-1 HB06 strain 28 days after immunization. Group 11 was the control group. All rabbits received intranasal administration of the *M. bovis* HB150, BoHV-1 gG-/tk-, and BoHV-1 HB06 strains using a 2 mL sterile syringe. The *M. bovis* HB0801 strain was introduced via tracheal administration. Each rabbit was kept in a separate housing to avoid the risk of cross-infection. The detailed information is summarized in Table 1.

### 2.3. Clinical Observation and Sample Collection

Clinical observation and rectal temperature were continuously recorded for 14 days after immunization and 14 days after the challenge. Animal weights were measured on day 0 before immunization, on day 28 before the challenge, and before euthanasia on day 56, which was 28 days after the challenge.

Nasal swabs were collected daily for 14 days postimmunization and postchallenge. Samples collected from *M. bovis* HB0801-challenged groups were placed and fully vortexed in tubes containing 1 mL sterile PBS, filtered through a 0.45 µm syringe filter; all samples were then stored at −20 °C for PCR/RT-PCR and *M. bovis* counting. Both serum and whole-blood samples were collected on days 0, 7, 14, 21, 28, 35, 42, 49, and 56 after immunization for antibody and cytokine detection. Lungs were collected and fixed in 4% paraformaldehyde for 48 h, then embedded in paraffin. Hematoxylin–eosin (HE) staining was performed on the sections, followed by histopathological examination.

### 2.4. Virus and Bacteria Shedding

Genomes of the treated nasal swabs were extracted, followed by PCR/RT-PCR to detect the *M. bovis* using the uvrC gene and BoHV-1 using gG and TK genes, as previously described [12,16]. The primers used in this study are listed in Table 2. The programs for *M. bovis* and BoHV-1 gG and tk genes are as follows:

*M. bovis* uvrC gene PCR reaction conditions: 95 °C for 3 min; 95 °C for 15 s, 55 °C for 15 s, 72 °C for 30 s, 35 cycles; 72 °C for 5 min. For the BoHV-1 gG PCR reaction conditions: 95 °C for 3 min; 95 °C for 15 s, 60.5 °C for 15 s, 72 °C for 40 s, 35 cycles; 72 °C for 5 min. Similarly, for the BoHV-1 tk gene PCR, the conditions were: 95 °C for 3 min; 95 °C for 15 s, 60.5 °C for 15 s, 72 °C for 30 s, 35 cycles; 72 °C for 5 min.

To assess the quantity of *M. bovis* HB0801 shedding following the challenge, the nasal swabs that had undergone treatment were subjected to 10-fold serial dilutions ranging from 10^8^. Subsequently, 100μL from each dilution was introduced into a PPLO liquid medium and incubated at 37℃ within a 5% CO_2_ incubator for a period of 3–5 days, or until there was no further change in the color of the medium. The highest dilution that resulted in a color change was designated as 1 CCU/mL. The *M. bovis* HB0801 shedding counting was then calculated according to the dilution.

To quantify the amount of BoHV-1 HB06 shedding after the challenge, the extracted DNA from the nasal swabs was used for the RT-PCR detection established in our lab using the gB gene. The program for the BoHV-1 gB gene was as follows: 95 °C for 30 s; 95 °C for 10 s, 60 °C for 20 s, 40 cycles; 95 °C for 15 s, 60 °C for 20 s, 95 °C for 15 s.

### 2.5. Neutralization Assay

Serum samples were collected weekly throughout the study. The serum samples were subjected to 56 °C for 30 min to inactivate any potential agents. Following inactivation, the serum was serially diluted in a 96-well cell culture plate and incubated with the BoHV-1 HB06 virus at a concentration of 100TCID_50_ in a 5% CO_2_ incubator at 37 °C for 1 h. The serum–virus mixture was introduced into a 96-well cell culture plate with MDBK cells. The plate was then incubated for three days at 37 °C in a 5% CO_2_ incubator. Neutralizing antibody titers were calculated using the Reed–Muench method, representing the highest serum dilution inhibiting the BoHV-1 virus infection.

### 2.6. Serum Antibody Level of M. bovis

*M. bovis* antibodies were determined by a competitive ELISA established in our lab. Briefly, the 4× diluted tested serum, positive and negative serum controls, and HRP-labeled monoclonal antibodies were added to the *M. bovis* p579-protein-coated plate and incubated at 37 °C for 60 min. After complete washing, 100 µL of the substrate chromogenic solution was added and incubated at room temperature in the dark for 10 min, and stop solution was added to stop the reaction. Values under the OD_450nm_ were taken. The blocking rate (PI value) was calculated according to the following calculation formula:

PI value = (1 − S/N) × 100%, where S = sample OD_450nm_; N = mean OD_450nm_ of the negative control serum. Conditions for the establishment of the test: 0.65 < OD_450nm_, negative control < 2.0, and PI positive control > 60%. PI sample ≥ 41% means positive; PI sample < 41% means negative.

### 2.7. Detection of Serum IFN-β and TNF-α

The serum samples were used to detect IFN-β and TNF-α using commercial ELISA kits (Ruixin Biotechnology Co., Ltd., Quanzhou, China) according to the manufacturers’ instructions. In brief, diluted serum samples were added to the plate wells and incubated at 37 °C for 30 min. After adequate washing, the secondary antibody was added; after being incubated at 37 °C for 30 min, the plate was washed sufficiently and substrate-color-development solution was added. The reaction was stopped and read under OD_450nm_.

### 2.8. Statistical Analysis

The *t*-test and a one-way ANOVA were used to compare the data of different groups; mean ± SEM was used. The comparison results of *p* < 0.05 (*), *p* < 0.01 (**), *p* < 0.001 (***), or *p* < 0.0001 (****) were considered to have significant or highly significant statistical differences.

## 3. Results

### 3.1. Clinical Symptoms

**Figure 1:**
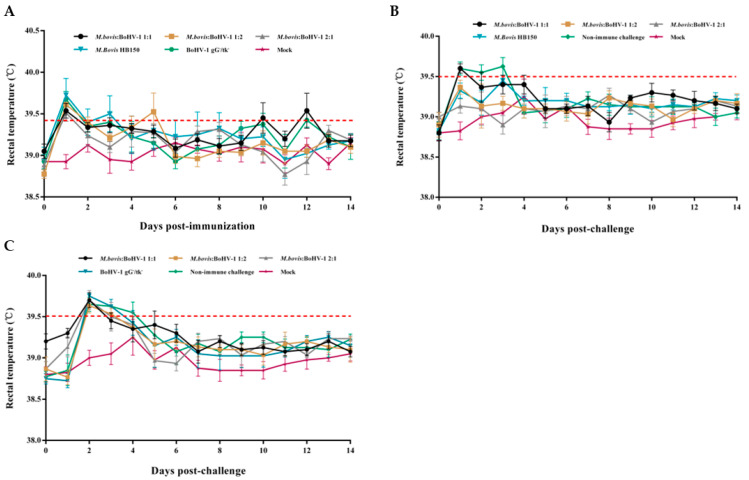
Rectal temperature of experimental rabbits pre- (**A**) and postchallenge by *M. bovis* HB0801 (**B**) and BoHV-1 HB06 (**C**).

**Figure 2:**
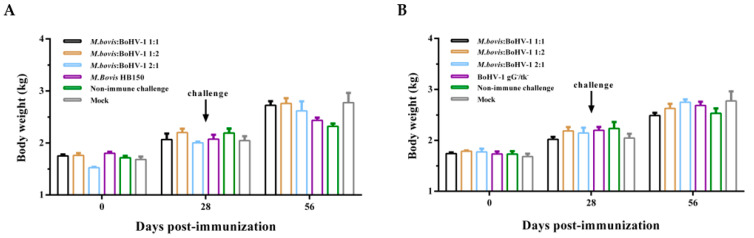
Changes in the mean body weight of the rabbits in the *M. bovis* groups (**A**) and BoHV-1 (**B**) groups during the entire experimental period.

Rectal temperature was slightly elevated one day after immunization, and returned to normal (Figure 1A). After the challenge with *M. bovis* HB0801 and BoHV-1 HB06, the rectal temperature reached higher than 39.5 °C from days 1–3 and 2–4, respectively, then returned to normal (Figure 1B,C). The body weight showed no significant difference after the immunization and challenge (Figure 2).

### 3.2. M. bovis HB150 and BoHV-1 gG-/tk- Detection after Immunization

*M. bovis* HB150 and BoHV-1 gG-/tk- were detected after immunization by using PCR. The immunization groups were categorized as follows: Groups 1 and 2 as a 1:1 ratio immunization group, Groups 3 and 4 as a 1:2 ratio immunization group, Groups 5 and 6 as a 2:1 ratio immunization group, Group 7 as a BoHV-1 gG-/tk- immunization group, Group 8 as a *M. bovis* HB150 immunization group, and Group 11 as the blank control.

Groups immunized with the antigen ratios of 1:2, 2:1, and *M. bovis* HB150 all stopped shedding on the sixth day after immunization. In the 1:1 and 1:2 groups, more than 50% of the animals exhibited continuous shedding on day 3 postimmunization. In contrast, in the 2:1 and *M. bovis* HB150 immunization groups, this condition persisted until day 4 postimmunization (Table 3).

The findings indicated that, on day 9 postimmunization, the 1:1, 2:1, and BoHV-1 gG-/tk- immunization groups did not detect the BoHV-1 gG-/tk- strain. The 1:2 group stopped shedding 8 days postimmunization. The control group showed no viral shedding throughout the observation period. On day 5 postimmunization, in the 1:1 and 1:2 groups, over 50% of the animals displayed continuous shedding, and more than 50% of the animals in the 2:1 and BoHV-1 gG-/tk- immunization groups were shedding by day 6 and day 7 postimmunization, respectively (Table 3).

These results suggest that the 2:1 mixed-immunization group induced the highest and most persistent levels of shedding of both pathogens in all mixed-immunization groups, followed by the 1:1 and 1:2 mixed-immunization groups.

### 3.3. M. bovis HB0801 and BoHV-1 HB06 Detection after Challenge

**Figure 3:**
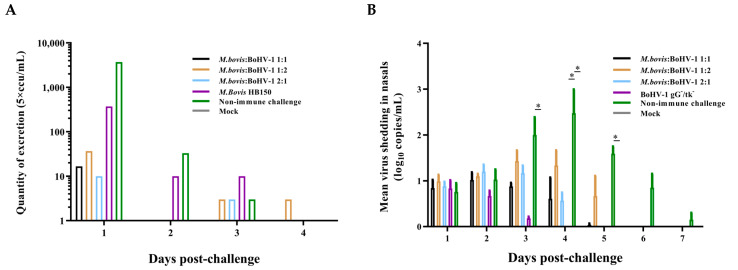
Detection of *M. bovis* HB0801 (**A**) and BoHV-1 HB06 (**B**) shedding in nasal swabs after the challenge. Nasal swabs were collected every day.

After the *M. bovis* HB0801 challenge, only a small amount of *M. bovis* HB0801 shedding could be detected in the 1:1 group on the first day. Mixed-vaccine-immunization and *M. bovis* HB150-immunized groups demonstrated lower *M. bovis* HB0801 shedding compared to the nonimmune-challenged group from day 1–2, but there was no statistical difference between the vaccinated groups and the nonimmune-challenged group (Figure 3A).

BoHV-1 HB06 shedding after the challenge was accessed using RT-PCR (Figure 3B). Importantly, no statistically significant variations were observed across all immunization groups throughout the observation period. Mixed-vaccine-immunization groups exhibited lower BoHV-1 HB06 shedding compared to the nonimmune-challenged group from day 3 to 7 postchallenge. The BoHV-1 gG-/tk- immunization group had significantly lower BoHV-1 HB06 shedding compared to the challenge-without-immunization group on day 4 postchallenge (*p* < 0.05). On day 3–5 postchallenge, animals challenged without immunization showed a considerably higher amount of BoHV-1 HB06 shedding than the blank control group (*p* < 0.05).

Taken together, the 1:1 group maintained the lowest shedding amount compared to all other mixed-immunization groups after the challenges of *M. bovis* HB0801 or BoHV-1 HB06.

### 3.4. Antibodies Test

**Figure 4:**
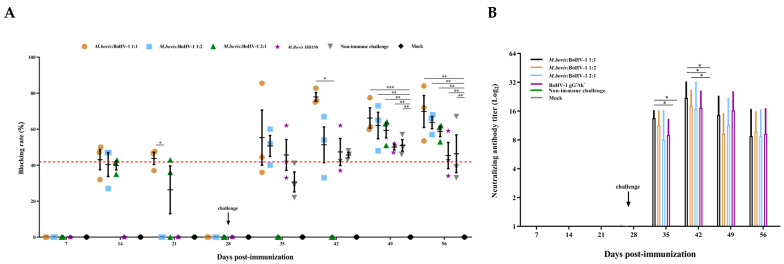
Humoral immune responses induced by the *M. bovis*–BoHV-1 bivalent vaccine. Serum samples were collected weekly to determine the (**A**) *M. bovis* serum antibody and (**B**) BoHV-1 serum neutralizing antibody. Variation is expressed as standard deviation.

To investigate the level of *M. bovis* serum antibodies after the immunization and challenge, competitive ELISA was used to detect the changes in serum. The antibody levels of the 1:1 group were significantly higher than those of the blank control group 21 days after immunization (*p* < 0.001). After the challenge, the highest titer was observed in the 1:1 group throughout the entire observation period. On day 7 postchallenge, the 1:1 immunized group displayed a significantly higher antibody titer compared to all other groups, including the immunized and nonimmunized groups (*p* < 0.05), and peaked at day 14 after the challenge (*p* < 0.05). From day 21 to 28 postchallenge, all immunized groups showed significantly higher antibody levels compared to the blank control group (*p* < 0.01) (Figure 4A).

The serum neutralization test was conducted to measure the level of BoHV-1 neutralizing antibodies in rabbits. All immunized groups showed minimal levels of the neutralization titer until 7 days postchallenge (35 days postimmunization). The 1:1 group induced a significantly higher neutralization titer compared to the challenge-without-immunization group at 7 and 14 days postchallenge (35 and 42 days postimmunization) (*p* < 0.05). The neutralization titer of the 1:2 group was significantly higher than that of the blank control 14 days postchallenge (*p* < 0.05) (Figure 4B).

In summary, we believe that the 1:1 mixed-immunization group can induce the highest and most prolonged *M. bovis* and BoHV-1 antibody titers among all immunization groups.

### 3.5. IFN-β and TNF-α Expression Detection

**Figure 5:**
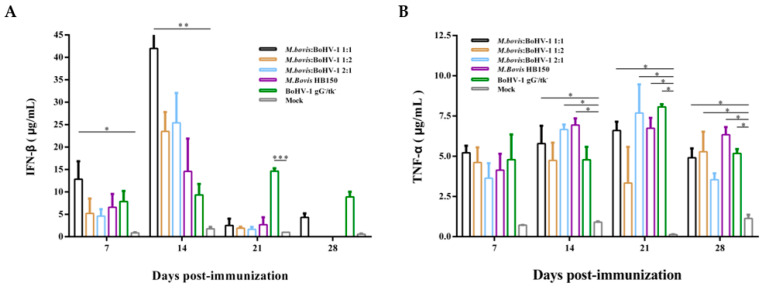
Production of IFN-β (**A**) and TNF-α (**B**) after immunization. The cytokines were detected with commercial ELISA kits. Results are shown as means ± SEM.

Generally, the expression of IFN-β was higher than that of the control group from day 7 to 21 postimmunization. At days 7 and 14 postimmunization, the 1:1 group exhibited significantly higher expression levels than the control group (*p* < 0.05 and *p* < 0.01, respectively). By day 21 postimmunization, a sharp decline was observed in all immunization groups, except for the BoHV-1 gG-/tk- single-vaccination group, which had significantly higher expression than the blank control (*p* < 0.001). At 28 days postimmunization, except for the 1:1 and BoHV-1 gG-/tk- single-immunization groups, the expression of IFN-β could not be detected in the other groups (Figure 5A).

TNF-α expression levels were consistently low in all experimental groups throughout the immunization period; however, the 1:1 and *M. bovis* HB150 immunization groups showed significantly higher expression levels than the blank control groups from day 14 to 28 postimmunization (*p* < 0.05). The 2:1 and 1:2 groups only induced higher TNF-α levels on days 14 and 28 postimmunization, respectively (*p* < 0.05). The BoHV-1 gG-/tk- single-vaccination group induced higher levels of TNF-α on days 21 and 28 postimmunization (*p* < 0.05) (Figure 5B).

The data above show that the 1:1 mixed group provoked a superior cellular immune response among all the immunized groups.

### 3.6. Histopathology Observation

**Figure 6:**
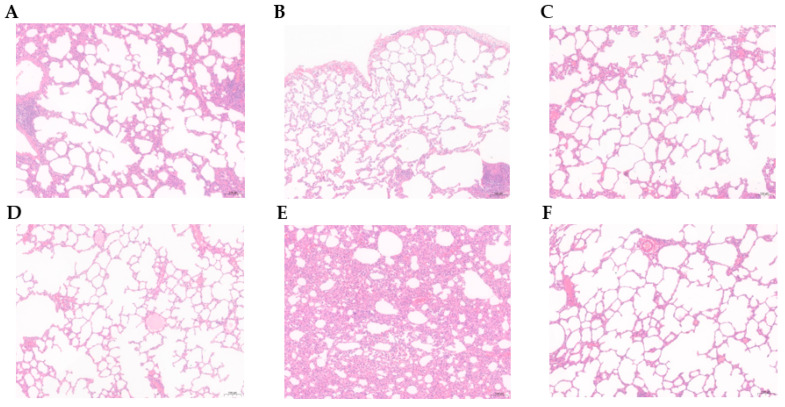
Histopathological images of lung tissues from the experimental rabbits stained by H&E. (**A**–**C**) represent experimental groups with different antigenic ratios of 1:1, 1:2, and 2:1, respectively. (**D**–**F**) represent experimental groups of the *M. bovis* HB150 immunization vaccine, the unvaccinated group, and the blank control group as the negative control.

According to the results, in the *M. bovis* HB0801-challenged groups, the alveolar structural morphology of the 1:1 and 1:2 groups were still intact, but the alveolar wall of the 1:1 group was thicker than that of the 2:1 group, and showed a small amount of plasmacytic exudation in the alveolar with inflammatory cells in the alveolar cavity (Figure 6A,B). The alveolar structure of the 2:1 group was relatively complete, with the entire alveolar wall and no inflammatory cell exudation (Figure 6C). The alveolar structure of the *M. bovis* HB150 single-component group remained intact, with no obvious pathological alterations in the alveolar wall and alveolar cavity (Figure 6D). In the nonimmunized-challenged group, the normal lung-tissue structure was disappeared, the alveolar wall was significantly thickened, and the alveoli became smaller or fused into a larger alveolar cavity with fewer alveoli; there were a large number of necrotic alveolar epithelial cells and inflammatory cell infiltration in the alveolar cavity, the bronchial mucosa epithelial hyperplasia, and venous stasis, and the lesion was the most severe (Figure 6E). All tissue from the blank control group exhibited a normal alveolar structure, with clean alveolar cavities and smooth alveolar walls, and no signs of inflammatory exudate were observed (Figure 6F).

**Figure 7:**
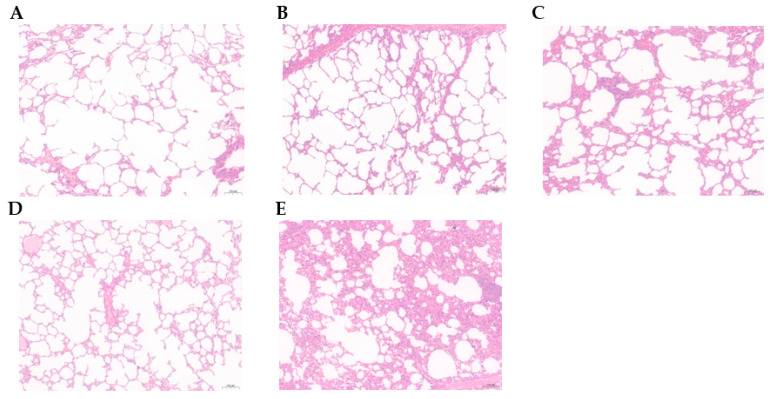
Histopathological images of lung tissues from the experimental rabbits stained by H&E. (**A**–**C**) represent experimental groups with different antigenic ratios of 1:1, 1:2, and 2:1, respectively. (**D**,**E**) represent experimental groups of the BoHV-1 gG-/tk- immunization vaccine and unvaccinated groups.

For the animals challenged with BoHV-1 HB06, the alveolar structures in the 1:1 and 1:2 groups remained mostly intact, with only a slight thickening of the alveolar wall and a small amount of plasmacytic detected in the alveolar cavity (Figure 7A,B). The alveolar structure of the 2:1 group was slightly damaged, and considerable alveolar epithelial cell proliferation was evident (Figure 7C). The BoHV-1 gG-/tk- immunization group had a complete alveolar structure and normal alveolar walls, but the interstitium was significantly congested (Figure 7D). In contrast, the nonimmune-challenged group’s alveolar structure was entirely destroyed, with extensively proliferated alveolar walls. Moreover, the alveolar cavity was replete with numerous necrotic alveolar epithelial cells and inflammatory cells, and the bronchial mucosal epithelium experienced necrosis and severe lesions (Figure 7E).

The results illustrate that the 2:1 and 1:2 groups provided the most significant protection among the mixed-immunization groups following the challenges with *M. bovis* HB0801 and BoHV-1 HB06. However, when considering all the mixed-immunization groups, the 1:1 group outperformed all others.

## 4. Discussion

In this study, we thoroughly evaluated the attenuated *M. bovis*–BoHV-1 bivalent vaccine using a rabbit model, and the results confirm its safety for overall health. Immunological analysis and challenge experiments showed that the *M. bovis*–BoHV-1 bivalent vaccine can elicit the antibodies against both *M. bovis* and BoHV-1, successfully protecting rabbits against live *M. bovis* and BoHV-1 challenges. The ratio of *M. bovis* HB150: BoHV-1 gG-/tk- 1:1 was highly recommended among all groups.

During the immunization and challenge experiment, rabbits were subjected to intranasal immunization with three different vaccine ratios, which induced protective antibodies and cytokines. All bivalent-vaccine groups generally demonstrated immune responses compared to the two single-vaccine groups. After the challenge, all experimental groups experienced a temporary elevation in rectal temperature for 1 day, except for the nonimmune-challenged group, which a sustained a high temperature for three consecutive days. All bivalent vaccines effectively shielded the rabbits from fever, aligning with our previous single-vaccine research studies [12,14].

The shedding of pathogens during the challenge period was a vital indicator of the disease [17]. For *M. bovis*, even early on day 2 postchallenge, the ratio of the 1:1 mixed vaccine could effectively suppress *M. bovis* HB0801 shedding, demonstrating superior protection compared to the single-vaccine-immunization group. Following 2 to 4 days of BoHV-1 incubation postchallenge, the virus replicated in the mucosal and tonsil tissues, leading to clinical signs in cattle and the subsequent shedding of the virus between days 3 and 10 following the initial infection [18]. Our study confirmed BoHV-1 shedding in the nasal by quantifying the viral titer. Although the BoHV-1 gG-/tk- single-vaccination group did not show noticeable shedding on day 4 postchallenge, there was no statistically significant difference among all the vaccinated groups, and the 1:1 mixed-vaccine group displayed deficient shedding levels on day 5. Nonimmune-challenged rabbits had continuous shedding until day 7.

We measured the antibody titers against *M. bovis* and BoHV-1, and the expression of cytokines such as IFN-β and TNF-α to assess the humoral and cell-mediated immunity responses related to protection. The 1:1 vaccine group initially exhibited the most significant increase in IFN-β and TNF-α postimmunization. Furthermore, after the challenge, animals vaccinated with the 1:1 ratio also displayed the highest antibody titers. Previous research reported that the gene-deletion vaccine can induce high antibody titers after the BoHV-1 challenge, consistent with our findings [19].

The stimulation of IFN-β transcription represents an early response to viral infection, with IFN-β expression exhibiting potent antiviral activity [20]. In this study, the 1:1 mixed vaccine induced the earliest and highest IFN-β expression compared to all other groups until 21 days after immunization, highlighting that 1.0 × 10^8^ CFU *M. bovis* HB150 combined with 1.0 × 10^6^ TCID_50_ BoHV-1 gG-/tk- led to the best immunoprotective response among all vaccine groups.

TNF-α is a proinflammatory cytokine that facilitates endothelial activation and the recruitment of leukocytes to infection sites [21]. It is one of the most important inflammatory mediators that plays a vital role in the initiation and pathogenesis of pulmonary fibrosis by inducing fibroblast proliferation, escalating collagen accumulation, and promoting apoptosis [22,23]. Based on the current study, although the immunized rabbits expressed a relatively low level of TNF-α, they exhibited significantly higher levels compared to the control group, indicating that all types of vaccines used in our study effectively induced proinflammatory immune responses.

Histopathological examination was also used to assess the protective efficacy. Generally, rabbits immunized with the 1:1, 1:2 mixed vaccines, and two single vaccines had nearly normal alveolar structures, while the nonimmune-challenged ones exhibited severe pulmonary pathological changes.

In summary, the *M. bovis*–BoHV-1 bivalent vaccine was safe and effective to rabbits, with the most effective combination being 1.0 × 10^8^ CFU *M. bovis* HB150 mixed with 1.0 × 10^6^ TCID_50_ BoHV-1 gG-/tk-. In addition to enhancing long-term immune responses, the bivalent vaccine also offered cellular protection against the *M. bovis* and BoHV-1 challenge. This model holds significant promise for potential application in cattle. However, further evaluation of bivalent vaccine efficacy in cattle remains an essential avenue for exploration.

## 5. Conclusions

Based on preliminary research, an attenuated *M. bovis*–BoHV-1 bivalent vaccine was developed for the first time. This study concluded that this vaccine elicited a robust production of serum antibodies and significantly elevated the production of both IFN-β and TNF-α, with the 1:1 antigen ratio having the best effect. These results suggest that the vaccine provides effective, adequate protection for the effective protection of rabbits, and lays the foundation for a series of subsequent experiments in cattle.

## Figures and Tables

**Table 1:** Animal immunization and challenge information.

Group	Vaccination Strain and Dose *	Challenge Strain and Dose **
1	1.0 × 10^8^ CFU *M. bovis* HB150 1.0 × 10^6^ TCID_50_ BoHV-1 gG-/tk-	4.0 × 10^7^ TCID_50_ BoHV-1 HB06
2	1.0 × 10^8^ CFU *M. bovis* HB150 1.0 × 10^6^ TCID_50_ BoHV-1 gG-/tk-	1.0 × 10^9^ CFU *M. bovis* HB0801
3	1.0 × 10^8^ CFU *M. bovis* HB150 2.0 × 10^6^ TCID_50_ BoHV-1 gG-/tk-	4.0 × 10^7^ TCID_50_ BoHV-1 HB06
4	1.0 × 10^8^ CFU *M. bovis* HB150 2.0 × 10^6^ TCID_50_ BoHV-1 gG-/tk-	1.0 × 10^9^ CFU *M. bovis* HB0801
5	2.0 × 10^8^ CFU *M. bovis* HB150 1.0 × 10^6^ TCID_50_ BoHV-1 gG-/tk-	4.0 × 10^7^ TCID_50_ BoHV-1 HB06
6	2.0 × 10^8^ CFU *M. bovis* HB150 1.0 × 10^6^ TCID_50_ BoHV-1 gG-/tk-	1.0 × 10^9^ CFU *M. bovis* HB0801
7	1.0 × 10^6^ TCID_50_ BoHV-1 gG-/tk-	4.0 × 10^7^ TCID_50_ BoHV-1 HB06
8	1.0 × 10^8^ CFU *M. bovis* HB150	1.0 × 10^9^ CFU *M. bovis* HB0801
9	DMEM medium	4.0 × 10^7^ TCID_50_ BoHV-1 HB06
10	Complete PPLO medium	1.0 × 10^9^ CFU *M. bovis* HB0801
11	Blank control	

* All vaccines were inoculated through the nasal cavity using a 2 mL sterile syringe. ** *M. bovis* HB0801 was challenged through tracheal injection; BoHV-1 HB06 was challenged through intranasal inoculation.

**Table 2:** Detailed primer sequences.

Target Gene	Primer Sequence (5′→3′)	Target Gene Length
gG	Forward: CCGACCGCCTCCTACACCAGATGCT	Delated strain: 524 bp Wild-type strain: 1859 bp
	Reverse: GGGTGTAGGCAAGCTCACCGCAACG	
TK	Forward: ACGGGCTGGGAAAGACAACAACGG	Delated strain: 235 bp Wild-type strain: 868 bp
	Reverse: GCGGACACGTCCAGCACGAACA	
gB	Forward: AGCACCTTTGTGGACCTAA	118 bp
	Reverse: GCTGTATCTCGCTGTAGTCG	
uvrC	Forward: TAATTTAGAAGCTTTAAATGAGCGC	238 bp
	Reverse: CATATCTAGGTCAATTAAGGCTTTG	

**Table 3:** Shedding of *M. bovis* HB150 and BoHV-1 gG-/tk- after immunization.

Group		Days Postimmunization (%)								
		1	2	3	4	5	6	7	8	9
1 and 2 (1:1)	*M. bovis*	6/6 (100)	4/6 (66.7)	4/6 (66.7)	1/6 (16.7)	0/6 111(0)	0/6 (0)	0/6 (0)	0/6 (0)	0/6 (0)
	BoHV-1	6/6 (100)	6/6 (100)	5/6 (83.3)	6/6 (100)	6/6 (100)	3/6 (50)	2/6 (33.3)	1/6 (16.7)	0/6 (0)
3 and 4 (1:2)	*M. bovis*	5/6 (83.3)	4/6 (66.7)	4/6 11(66.7)	2/6 (33.3)	2/6 (33.3)	0/6 (0)	0/6 (0)	0/6 (0)	0/6 (0)
	BoHV-1	6/6 (100)	6/6 (100)	6/6 (100)	5/6 (83.3)	5/6 (83.3)	3/6 (50)	1/6 (16.7)	0/6 (0)	0/6 (0)
5 and 6 (2:1)	*M. bovis*	6/6 (100)	5/6 (83.3)	6/6 (100)	4/6 11(66.7)	2/6 (33.3)	0/6 (0)	0/6 (0)	0/6 (0)	0/6 (0)
	BoHV-1	6/6 (100)	6/6 (100)	5/6 (83.3)	6/6 (100)	6/6 (100)	4/6 11(66.7)	3/6 (50)	1/6 (16.7)	0/6 (0)
7 (BoHV-1 gG-/tk-)		3/3 (100)	3/3 (100)	3/3 (100)	2/3 (66.7)	3/3 (100)	3/3 (100)	2/3 (66.7)	1/3 (33.3)	0/3 (0)
8 (*M. bovis* HB150)		3/3 (100)	3/3 (100)	3/3 (100)	2/3 (66.7)	1/3 (33.3)	0/3 (0)	0/3 (0)	0/3 (0)	0/3 (0)
11 (mock)		0/3	0/3	0/3	0/3	0/3	0/3	0/3	0/3	0/3

## Data Availability

All data during the current study are available from the corresponding author upon making a reasonable request.

## References

[1] Blakebrough-Hall C., McMeniman J.P., Gonzalez L.A. (2020). An evaluation of the economic effects of bovine respiratory disease on animal performance, carcass traits, and economic outcomes in feedlot cattle defined using four BRD diagnosis methods. J. Anim. Sci..

[2] Cusack P.M.V. (2023). Evaluation of practices used to reduce the incidence of bovine respiratory disease in Australian feedlots (to November 2021). Aust. Vet. J..

[3] Windeyer M.C., Leslie K.E., Godden S.M., Hodgins D.C., Lissemore K.D., LeBlanc S.J. (2014). Factors associated with morbidity, mortality, and growth of dairy heifer calves up to 3 months of age. Prev. Vet. Med..

[4] Gandhi N.N., Inzana T.J., Rajagopalan P. (2023). Bovine Airway Models: Approaches for Investigating Bovine Respiratory Disease. ACS Infect. Dis..

[5] Dudek K., Szacawa E. (2020). Mycoplasma bovis Infections: Occurrence, Pathogenesis, Diagnosis and Control, Including Prevention and Therapy. Pathogens.

[6] Guo T., Zhang J.H., Chen X.D., Wei X., Wu C.X., Cui Q., Hao Y.Q. (2021). Investigation of viral pathogens in cattle with bovine respiratory disease complex in Inner Mongolia, China. Microb. Pathog..

[7] McGill J.L., Sacco R.E. (2020). The Immunology of Bovine Respiratory Disease Recent Advancements. Vet. Clin. N. Am.-Food A.

[8] Nicola I., Cerutti F., Grego E., Bertone I., Gianella P., D’Angelo A., Peletto S., Bellino C. (2017). Characterization of the upper and lower respiratory tract microbiota in Piedmontese calves. Microbiome.

[9] Cowick C.A., Russ B.P., Bales A.R., Nanduri B., Meyer F. (2022). Mannheimia haemolytica Negatively Affects Bovine Herpesvirus Type 1.1 Replication Capacity In Vitro. Microorganisms.

[10] Oliveira T.E.S., Pelaquim I.F., Flores E.F., Massi R.P., Valdiviezo M.J.J., Pretto-Giordano L.G., Alfieri A.A., Saut J.P.E., Headley S.A. (2020). Mycoplasma bovisand viral agents associated with the development of bovine respiratory disease in adult dairy cows. Transbound. Emerg. Dis..

[11] Qi J.J., Guo A.Z., Cui P., Chen Y.Y., Mustafa R., Ba X.L., Hu C.M., Bai Z.D., Chen X., Shi L. (2012). Comparative Geno-Plasticity Analysis of Mycoplasma bovis HB0801 (Chinese Isolate). PLoS ONE.

[12] Zhang R., Han X.X., Chen Y.Y., Mustafa R., Qi J.J., Chen X., Hu C.M., Chen H.C., Guo A.Z. (2014). Attenuated Mycoplasma bovis strains provide protection against virulent infection in calves. Vaccine.

[13] Chen X.L., Wang X., Qi Y.P., Wen X.B., Li C.X., Liu X.B., Ni H.B. (2018). Meta-analysis of prevalence of bovine herpes virus 1 in cattle in Mainland China. Acta Trop..

[14] Zhang M.M., Fu S.L., Deng M.L., Xie Q., Xu H.Y., Liu Z.F., Hu C.M., Chen H.C., Guo A.Z. (2011). Attenuation of bovine herpesvirus type 1 by deletion of its glycoprotein G and tk genes and protection against virulent viral challenge. Vaccine.

[15] Chao J., Han X.X., Liu K., Li Q.N., Peng Q.J., Lu S.Y., Zhao G., Zhu X.F., Hu G.Y., Dong Y.Q. (2019). Calves Infected with Virulent and Attenuated Mycoplasma bovis Strains Have Upregulated Th17 Inflammatory and Th1 Protective Responses, Respectively. Genes.

[16] Marawan M.A., Deng M.L., Wang C., Chen Y.Y., Hu C.M., Chen J.G., Chen X., Chen H.C., Guo A.Z. (2021). Characterization of BoHV-1 gG-/tk-/gE- Mutant in Differential Protein Expression, Virulence, and Immunity. Vet. Sci..

[17] Chung Y.C., Shen H.Y., Cheng L.T., Liu S.S., Chu C.Y. (2016). Effectiveness of a BHV-1/BEFV bivalent vaccine against bovine herpesvirus type 1 infection in cattle. Res. Vet. Sci..

[18] Salt J.S., Thevasagayam S.J., Wiseman A., Peters A.R. (2007). Efficacy of a quadrivalent vaccine against respiratory diseases caused by BHV-1PI(3)V, BVDV and BRSV in experimentally infected calves. Vet. J..

[19] Belknap E.B., Walters L.M., Kelling C., Ayers V.K., Norris J., McMillen J., Hayhow C., Cochran M., Reddy D.N., Wright J. (1999). Immunogenicity and protective efficacy of a gE, gG and US2 gene-deleted bovine herpesvirus-1 (BHV-1) vaccine. Vaccine.

[20] da Silva L.F., Jones C. (2011). Infection of cultured bovine cells with bovine herpesvirus 1 (BHV-1) or Sendai virus induces different beta interferon subtypes. Virus Res..

[21] Gondaira S., Higuchi H., Iwano H., Nakajima K., Kawai K., Hashiguchi S., Konnai S., Nagahata H. (2015). Cytokine mRNA profiling and the proliferative response of bovine peripheral blood mononuclear cells to Mycoplasma bovis. Vet. Immunol. Immunopathol..

[22] Kiraz Y., Adan A., Yandim M.K., Baran Y. (2016). Major apoptotic mechanisms and genes involved in apoptosis. Tumor Biol..

[23] Bringardner B.D., Baran C.P., Eubank T.D., Marsh C.B. (2008). The role of inflammation in the pathogenesis of idiopathic pulmonary fibrosis. Antioxid. Redox Sign.

